# Traditional risk factors for essential hypertension: analysis of their specific combinations in the EPIC-Potsdam cohort

**DOI:** 10.1038/s41598-019-38783-5

**Published:** 2019-02-06

**Authors:** Violetta Andriolo, Stefan Dietrich, Sven Knüppel, Wolfgang Bernigau, Heiner Boeing

**Affiliations:** 1 Department of Epidemiology, German Institute of Human Nutrition, Potsdam-Rehbrücke (DIfE), Germany; 2 Department of Molecular Epidemiology, German Institute of Human Nutrition, Potsdam-Rehbrücke (DIfE), Germany

## Abstract

Appropriate interventions might improve the prevention of essential hypertension. This requires a comprehensive view of modifiable lifestyle factors (MLFs) distribution and effect. To determine how six MLFs (general adiposity, abdominal adiposity, alcohol consumption, smoking, diet, physical inactivity) for risk of hypertension are distributed and how their combinations affect the risk, a prospective study cohort of 11,923 healthy participants from the population-based European Prospective Investigation into Cancer and Nutrition (EPIC)–Potsdam Study was used. Of these, 1,635 developed hypertension during a mean follow-up of 10.3 years. Mutually exclusive combinations, clustering and interactions of MLFs were then investigated stratifying by sex, Hazard Ratios (HRs) and Population Attributable Risks (PARs%) were calculated. General adiposity alone was sufficient to increase the risk of hypertension (HR = 1.86, PAR% 3.36), and in this cohort it played a major role in enhancing the risk of hypertension, together with smoking and physical inactivity. MLFs had a different impact and a different modulation of risk in women and men, and they showed a remarkable tendency to occur in specific patterns with higher prevalence than expected. This indication can help to promote a holistic approach through multifactorial preventive strategies addressing more than a factor at a time. For prevention of hypertension addressing adiposity together with smoking, promoting at the same time physical activity should be the first choice.

## Introduction

Despite massive improvements achieved in primary and secondary prevention, incidence and prevalence of essential hypertension (hereafter referred to as hypertension) are still rising worldwide, and further increases are foreseen if no effective counteractions are undertaken^1,2^. Population-based and/or individual interventions could be effective counteractions as they have shown to reduce blood pressure values and hypertension incidence^3,4^ and subsequently the risk of major cardiovascular diseases^5–7^. The implementation of interventions requires a good understanding of modifiable lifestyle factors (MLFs) within the targeted population^8,9^. This should also include insights on how the MLFs interact with each other and which MLFs profile could be a target of interventions with the highest potential impact.

So far the largest body of literature analysed MLFs separately, and information about how they are combined in the population and how such combinations impact risk are scarce, as well as information about clusters of association and mutual interactions. We identified six MLFs with sufficient evidence of a causal co-responsibility regarding the occurrence of hypertension: general and abdominal obesity^10–12^, excessive use of alcohol^13,14^, smoking^15,16^, lack of physical activity^17^ and low adherence to the DASH (Dietary Approaches to Stop Hypertension) diet^18,19^. Therefore, this study aimed to provide information about the prevalence and risk association of the MLFs combinations observed in a large adult German cohort, with insights about clustering and interactions of MLFs.

## Methods

### Study Design and population

The present study was performed in the prospective European Prospective Investigation into Cancer and Nutrition (EPIC)-Potsdam cohort. The ethical committee of the state of Brandenburg, Germany, approved the methods of the EPIC Potsdam Study, following the principles of the Helsinki Declaration for human rights. Informed consent was obtained from all the participants before their recruitment^20^. Data collection at baseline examination included self-administered questionnaires, interviews and physical examinations^21^. Diet was assessed using a food frequency questionnaire (FFQ). Details about the modality, validity and reproducibility of the FFQ have been previously published^22^. Data on anthropometric variables and blood pressure were obtained during physical examinations^21,23,24^. All interviews and examinations were conducted by trained interviewers who were regularly supervised^24^.

### Exclusion and inclusion criteria

**Figure 1:**
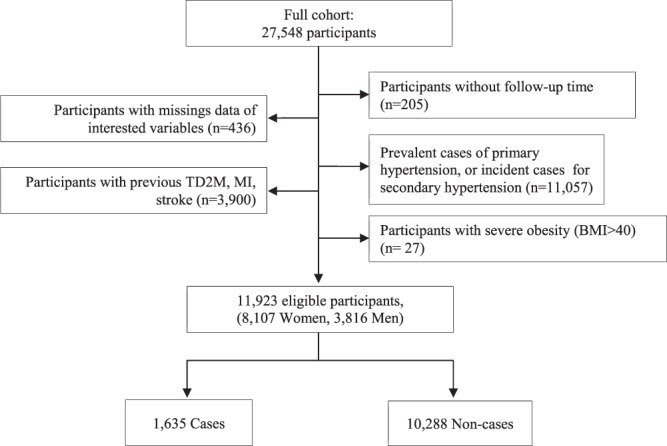
Flowchart of exclusions TD2M = Diabetes mellitus type 2; MI = myocardial infarction.

The entire study population included 27,548 participants aged 40–65 (men) and 35–65 (women). Participants with prevalent primary or secondary hypertension (n = 11,057), with no follow-up time (n = 205), or with missing values in any of the variables used (n = 436) were excluded (Fig. 1). Participants with prevalent chronic diseases (T2DM and cancer), previous cardiovascular or cerebrovascular disorders (myocardial infarction and stroke) (n = 3,689), or morbid obesity (BMI > 40 kg m^−2^) (n = 27) were also excluded, to avoid bias due to comorbidities. After exclusion, 11,923 participants (8,107 women and 3,816 men) were eligible for analysis. Of these, 1,635 (1,046 women and 589 men) developed hypertension during a mean (SD) follow-up time of 10.3(2.6) years.

### Definition and ascertainment of prevalent and incident hypertension

Prevalent cases are defined as participants with (i) systolic BP ≥ 140 mmHg and/or diastolic BP ≥ 90 mmHg as determined by the mean of the second and third measurements, (ii) self-reported prevalent hypertension diagnosis, or (iii) use of antihypertensive medication. Self-administered questionnaires were used every 2 to 3 years to collect information about potential cases of incident hypertension and hypertension-specific medicines during the previous months. Incident cases were included in the analysis only after verification and the confirmation of the diagnosis by the treating physician (*International Classification of Diseases Tenth Revision*: I10)^25^.

### Definition and categorisation of MLFs

Six acknowledged MLFs were selected: general adiposity, abdominal adiposity, physical activity, adherence to “Dietary Approach to Stop Hypertension” (DASH) diet, smoking, and alcohol consumption. Body Mass Index (BMI, kg/m²) and waist circumference accounted for general and abdominal adiposity, respectively. BMI was categorised as underweight/normal (<25), overweight (25–30) and obese (>30). Waist circumference was measured in cm and sex-specifically categorised as no risk (women < 80; men < 94), medium risk (women 80–88; men 94–102), and high risk (women > 88; men > 102). Smoking behaviour was categorised as never smoking, former and current smoking. The Improved Physical Activity Index (IPAI) adjusted for sex and age was used to account for physical activity; it was calculated as described in previous papers^26^ and divided into inactive, moderately active, and active/very active categories. Dietary intake data assessed by FFQ^22^ were used to calculate a DASH Diet Score^27^, that was modified due to lack of information about sodium intake. The modified DASH Score (mDASH) was divided into tertiles (low, medium and high) indicating adherence to the DASH diet. Alcohol intake from beverages was categorized as follows: no/light consumption (women 0–6 g/day; men 0–12 g/day), moderate (women > 6–12 g/day; men ≥ 12–24 g/day), high (women > 12–30 g/day; men > 24–60 g/day), and very high consumption (women > 30 g/day; men > 60 g/day). Based on previous knowledge, the following variables were used as adjusting variables in the risk models: educational attainment, age, and use of anti-inflammatory drugs. Educational attainment was categorised into high, medium, and low.

### Statistical Analysis

Baseline characteristics were presented as means and standard deviations for continuous variables and as frequencies for categorical variables. First, the selected MLFs were analysed in the initial categories for their association with incidence of hypertension using the category with the assumed lowest risk as a reference. Hazard ratios (HRs) and 95% confidence intervals (CIs) were computed using Cox proportional hazards regression stratified by age. Entry time was defined as the participant’s age at recruitment (years) and exit time as age at either diagnosis of hypertension, death or return of the last follow-up questionnaire. In the subsequent analyses, to calculate the population attributable risk (PAR%), MLFs were dichotomised as *risk/no risk*, according to the findings from the initial analysis. The *no risk* category was used as a reference when calculating the HR. PAR% was calculated assuming a causal and independent relationship between the MLF and incident hypertension^28^. The following equation was used:

$${\rm{PAR}} \% =100\times [({\rm{HR}}-1)\times \,{{\rm{P}}}_{{\rm{e}}}]/[({\rm{HR}}-1)\times \,{{\rm{P}}}_{{\rm{e}}}]+1],$$

where P_e_ is the exposed proportion^29^. Further, mutually exclusive combinations of MLFs were investigated, using the binary system of presence (=1)/absence (=0) of the dichotomised MLFs. The prevalence of each combination was assessed, and the HRs calculated. The PAR% of each combination was also determined.

Finally, the ratio between observed and expected (O/E) prevalence of each combination was calculated according to a method previously published^30,31^. The expected prevalence for each combination assuming the factors aggregate by chance was computed by multiplying the individual probabilities of each factor based on their observed prevalence, as shown in the example below:

$${\rm{P}}\,(1+3+4)={\rm{P1}}\,\times \,(1-{\rm{P2}})\,\times \,{\rm{P}}3\,\times \,{\rm{P}}4\,\times \,(1-{\rm{P}}5)$$

P1 to P5 represent the prevalence of the single factor observed in the whole study sample. 1 − P2 or 1 − P5 represents the complementary probability for the subject having the correspondent risk factor. We considered indicative of clustering an O/E prevalence ratio >1.

In addition, the risk performance of a combination was evaluated by comparing the observed HR with the expected HR assuming no interaction between factors. The expected HR was calculated by multiplying the HRs of each MLF:

$${{\rm{HR}}}_{{\rm{expected}}}(1+3+4)={\rm{HR}}1\,\times \,{\rm{HR}}\,3\,\times \,{\rm{HR}}4.$$

Interaction of MLFs occurs when their observed HR differs from the one expected to result from their individual effect.

We considered indicative of synergistic interaction an O/E HR ratio >1.

All analyses were performed with SAS software, version 9.4, and SAS Enterprise Guide, version 6.1 (SAS Institute Inc., Cary, NC, USA).

## Results

The baseline characteristics of the cohort are shown in Table 1. Participants who developed hypertension were older and more likely to be men and physically inactive, they had a higher BMI, waist circumference and slightly higher blood pressure at baseline. Men who developed hypertension but not women were more likely to be current or former smokers and to consume more than 24 g/day of alcohol. No appreciable difference was noticed in the DASH score between cases and non-cases in both sexes (Table 1).

**Table 1:** Baseline General Characteristics of Participants.

	Women (n = 8107)		MEN (n = 3816)	
	Cases of incident hypertension (n = 1046)	Non-cases (n = 7061)	Cases of incident hypertension (n = 589)	Non-cases (n = 3227)
Age, y (SD)	50.1(9.1)	45.6(8.6)	52.2 (7.48)	49.5 (7.6)
Follow-up time, y (SD)	6.4 (2.9)	10.8 (1.8)	6.5(2.9)	10.7(2)
BMI, Kg/m^2^ (SD)	26.1 (3.9)	24 (3.5)	26.8 (2.9)	25.4 (2.9)
Waist, cm (SD)	81.4 (10.5)	76.2 (9)	94.4 (8.5)	90.1 (8.6)
Systolic BP, mmHg (SD)	123.5 (8.6)	115.3 (9.8)	127.5 (7.7)	122.5 (9)
Diastolic BP, mmHg (SD)	80.6 (5.8)	76.1(6.8)	82.6 (5.8)	79.5 (6.2)
mDASH, score	14.6 (4.4)	14.6 (4.3)	12.3 (4.1)	12.3(4.3)
Smoking:				
Never (%)	608 (7.50)	3,861 (47.63)	171 (4.48)	1,147 (30.06)
Former (%)	230 (2.84)	1,781 (21.97)	243 (6.37)	1,196 (31.34)
Current (%)	208 (2.57)	1,419 (17.50)	175 (4.59)	884 (23.17)
Alcohol consumption:				
No/ light consumers (%)	169 (2.08)	1,056 (13.03)	44 (1.15)	303 (7.94)
Moderate consumers (%)	676 (8.34)	4,463 (55.05)	329 (8.62)	1,806 (47.33)
Heavy consumers (%)	162 (2)	1,263 (15.58)	178 (4.66)	956 (25.05)
Very heavy consumers (%)	39 (0.48)	279 (3.44)	38 (1)	162 (4.25)
Physical activity:				
Inactive (%)	290 (3.58)	1,447 (17.85)	168 (4.40)	694 (18.19)
Moderate (%)	431 (5.32)	2,887 (35.61)	221 (5.79)	1,300 (34.07)
Active/very act. (%)	325 (4.01)	2,727 (33.64)	200 (5.24)	1,233 (32.31)

A multivariable-adjusted model was built to test the thresholds of the risk of hypertension in a preliminary analysis (data not shown). Alcohol was the only MLF that was not associated with the incidence of hypertension and was therefore excluded from the subsequent analyses.

According to the finding of the preliminary analysis, the MLFs were dichotomised and the categories characterised by higher risk of hypertension were defined as follows: BMI > 25 Kg/m^2^, waist circumference >88 cm for women and >102 cm for men, lowest tertile of mDASH score, current and former smoking, and “inactive” category of IPAI index.

In the dichotomised, multivariable-adjusted model (Table 2) the HRs of the category at risk compared to the one at no risk were in accordance with the results of the previous analysis. The HR (95% CI) for incident hypertension was 1.80 (1.56–2.07) in women and 1.78 (1.47–2.16) in men for a BMI > 25. The estimated HR (95% CI) for high-risk waist circumference was 1.42 (1.20–1.67) in women and 1.59 (1.28–1.97) in men. For low adherence to the DASH diet, the risk appeared to be higher (HR 1.47, 95% CI 1.19–1.82) in women than in men (HR 1.02, 95% CI 0.81–1.23) compared to higher adherence. Smoking had a HR (95% CI) of 1.07 (1.01–1.18) compared to non-smoking, and the risk was higher in men (HR 1.20, 95% CI 1.01–1.45) than in women (HR1.04, 95% CI 0.91–1.18). Inactivity showed a HR (95% CI) of 1.15 (1.02–1.31) in women and of 1.16 (1.01.1.36) in men compared to active/very active participants. The estimated PARs, representing the percentage of cases attributable to the presence of risk factors, were in women and men 39.3 and 50.3% for high BMI, 14.9 and 14.6% for high risk waist circumference, 10.5 and 16.6% for low adherence to DASH diet, 31.8 and 44.2% for smoking, 34.2 and 34.6% for low physical activity.

**Table 2:** Hazard Ratios (CI 95%) and hypothesized Population Attributable Risks (PAR%) of hypertension in relation to single MLFs in men and women of the EPIC- POTSDAM cohort.

MLF	Men				Women			
	No. (%)	HR (95% CI)	aHR (95% CI)	PAR%	No. (%)	HR (95% CI)	aHR (95% CI)	PAR%
**BMI**								
No risk (<25)	1,646 (43.13)	1.0 (reference)	1.0 (reference)		5,187 (63.98)	1.0 (reference)	1.0 (reference)	
High risk (≥25)	2,170 (56.87)	2.01 (1.67–2.41)	1.78 (1.47–2.16)	50.37	2,920 (36.02)	2.03 (1.79–2.31)	1.80 (1.56–2.07)	39.35
**Waist**								
Below the limit	3,405 (89.23)	1.0 (reference)	1.0 (reference)		7,104 (87.63)	1.0 (reference)	1.0 (reference)	
Above the limit	411 (10.77)	2.06 (1.68–2.54)	1.59 (1.28–1.97)	14.62	1,003 (12.37)	2.01 (1.73–2.33)	1.42 (1.20–1.67)	14.97
**Dash Diet**								
Medium/high adherence	3,055 (80.06)	1.0 (reference)	1.0 (reference)		7,464 (92.07)	1.0 (reference)	1.0 (reference)	
Low adherence	761 (19.94)	1.01 (0.81–1.24)	1.02 (0.81–1.23)	16.65	643 (7.93)	1.42 (1.15–1.76)	1.47 (1.19–1.82)	10.46
**Smoking**								
Never	1,318 (34.54)	1.0 (reference)	1.0 (reference)		4,469 (55.13)	1.0 (reference)	1.0 (reference)	
Former/current	2,498 (65.46)	1.29 (1.07–1.54)	1.20 (1.01–1.45)	44.17	3,638 (44.87)	1.06 (0.94–1.21)	1.04 (0.91–1.18)	31.82
**Physical Activity**								
Moderate/Active/Very active	2,070 (54.25)	1.0 (reference)	1.0 (reference)		4,468 (55.11)	1.0 (reference)	1.0 (reference)	
Inactive	1,746 (45.75)	1.23 (1.05–1.45)	1.16 (1.01.1.36)	34.64	3,639 (44.89)	1.22 (1.08–1.39)	1.15 (1.02–1.31)	34.17

HR: adjusted for educational attainment, age and use of anti-inflammatory drugs;

aHR: HR adjusted for all the other MLFs;

BMI: *no risk*: Body Mass Index <25 kg/m²; *high risk*: ≥25 kg/m²;

WAIST: *below the limit*: waist circumference <88 cm women; <102 cm men; *above the limit*: ≥ 88women; ≥ 102men

DASH DIET: “Dietary Approach to Stop Hypertension” diet; *medium/high adherence:* DASH score 9–28; *low adherence*: DASH score 0–8.

### Combination of MLFs: prevalence, strength of association and PAR%

For simplicity of the combinations’ description we will use the following terms for the above-mentioned MLFs: BMI > 25: “BMI”; high-risk waist circumference: “waist”; low adherence to DASH diet: “diet”, former and current smoking: “smoking”; low physical activity: “inactivity”.

The analyses of specific combinations of MLFs revealed that a total of 1,580 women (19%) and 307 men (8%) were not exposed to any of the considered MLFs. (Tables 3–4). This group at no-risk was used as a reference. 2,885 women (35.6%) and 1,011 men (26.5%) were exposed to one single MLF only (Tables 3 and 4). The most common MLFs were smoking (15%women; 12%men), inactivity (12%women; 5%men), and BMI (7%women, 7%men). In this category, solely BMI showed an important association with the incidence of hypertension, with a HR of 1.78 (1.37–2.32) in women and 2.43 (1.43–4.13) in men (Tables 3 and 4).

**Table 3:** HR (CI 95%) and PAR% for hypertension in relation to specific combinations of MLFs, Men.

Combinations of mlfs	No. of cases among exposed (%)	Prevalence (%)	aHR (95% CI)	PAR %
**No MLF**	20 (6, 5)	307 (8.05)		
1 MLF	111 (11)	1, 011 (26.5)		
BMI	45 (15, 5	290 (7, 6)	2.43 (1.43–4.13)*	4.47
Waist	0	1 (0, 03)		
Diet	3 (5)	61 (1, 6)	0.83 (0.24–2.81)	
Smoking	44 (9, 6)	458 (12)	1.62 (0.95–2.75)	
Inactivity	19 (9, 5)	201 (5, 3)	1.55 (0.82–2.90)	
2 MLFs	193 (15)	1, 288 (33)		
BMI Inactivity	45 (19, 6)	230 (6)	3.09 (1.82–5.25)*	4.08
BMI Smoking	82 (16, 6)	494 (12, 9)	2.70 (1.66–4.42)*	8.16
BMI Waist	8 (18, 6)	43 (1, 1)	2.93 (1.28–6.69)*	0.74
BMI Diet	3 (7, 1)	42 (1, 1)	1.17 (0.35–3.97)	
Inactivity Diet	5 (11, 4)	44 (1, 1)	2.03 (0.75–5.44)	
Inactivity Smoking	40 (12, 5)	320 (8, 4)	1.95 (1.14–3.35)*	4.10
Diet Smoking	10 (8, 7)	115 (3)	1.63 (0.76–3.50)	
3 MLFs	379 (43, 4)	874 (22, 9)		
BMI Smoking Inactivity	86 (20, 3)	424 (11, 1)	3.13 (1.92–5.10)*	7.56
BMI Waist Smoking	41 (37, 3)	110 (2, 9)	7.18 (4.19–12.32)*	2.48
BMI Diet Inactivity	10 (23, 3)	43 (1, 1)	3.95 (1.84–8.48)*	0.84
BMI Diet Smoking	20 (18)	111 (2, 9)	3.28 (1.76–6.12)*	2.02
BMI Waist Diet	1 (20)	5 (0, 1)	3.57 (0–47–26.90)	
BMI Waist Inactivity	10 (23, 8)	42 (1, 1)	3.85 (1.79–8.27)*	0.81
Diet Smoking Inactivity	18 (12, 9)	139 (3, 6)	2.15 (1.13–4.08)*	1.95
4 MLFs	68 (22, 4)	303 (7, 9)		
BMI Diet Smoking Inactivity	22 (17, 5)	126 (3, 3)	2.95 (1.61–5.43)*	2.18
BMI Waist Smoking Inactivity	36 (26, 7)	135 (3, 5)	4.07 (2.35–7.05)*	2.66
BMI Waist Diet Inactivity	2 (22, 2)	9 (0, 2)	4.22 (0.97–18.29)	
BMI Waist Diet Smoking	8 (24, 2)	33 (0, 9)	3.96 (1.73–9.05)*	0.64
5 MLFs	11 (33, 3)	33 (0, 9)		
Waist Diet BMI Smoking Inactivity	11 (33, 3)	33 (0, 9)	5.72 (2.71–12.08)*	0.71
Total	589 (15, 4)	3, 816 (100)		

aHR: HR adjusted for all the other combination of MLFs;

*p < 0,05.

**Table 4:** HR (CI 95%) and PAR% for hypertension in relation to specific combinations of MLFs, Women.

Combinations of mlfs	No. of cases among exposed (%)	Prevalence (%)	HRa (95% CI)	hPAR %
**No MLF**	134 (8, 48)	1, 580 (19, 5)		
1 MLF	292 (10, 1)	2, 885 (35, 6)		
BMI	96 (16, 6)	579 (7, 1)	1.78 (1.37–2.32)*	3.14
Waist	2 (22, 2)	9 (0, 1)	2.44 (0.60–9.93)	
Diet	10 (10, 8)	93 (1, 1)	1.45 (0.76–2.77)	
Smoke	84 (6, 9)	1221 (15, 1)	0.95 (0.72–1.25)	
Inactivity	100 (10, 2)	983 (12, 1)	1.08 (0.83–1.40)	
2 MLFs	616 (26, 6)	2, 315 (28, 6)		
BMI Inactivity	106 (20)	530 (6, 5)	2.04 (1.57–2.64)*	3.34
BMI Smoke	49 (12, 6)	388 (4, 8)	1.53 (1.10–2.13)*	1.66
BMI Waist	49 (22, 9)	214 (2, 6)	2.41 (1.72–3.36)*	1.54
BMI Diet	7 (25)	28 (0, 3)	2.52 (1.15–5.47)*	0.21
Waist Diet	0	/1 (0, 01)		
Waist Inactivity	2 (33, 3)	6 (0, 1)	4.29 (1.05–17.52)*	0.06
Waist Smoke	0	4 (0, 05)		
Inactivity Diet	15 (12, 5)	120 (/1, 5)	1.51 (0.88–2.58)	
Inactivity Smoke	87 (9, 5)	916 (11, 3)	1.19 (0.91–1.57)	
Diet Smoke	9 (8, 3)	108 (1, 3)	1.20 (0.61–2.36)	
3 MLFs	213 (21, 3)	998 (12, 3)		
BMI Smoke Inactivity	59 (18, 4)	321 (4)	2.17 (1.59–2.95)*	2.14
BMI Waist Smoke	45 (22, 7)	198 (2, 4)	3.09 (2.19–4.33)*	1.65
BMI Diet Inactivity	10 (25)	40 (0, 5)	2.97 (1.55–5.66)*	0.33
BMI Diet Smoke	7 (31, 8)	22 (0, 3)	6.40 (2.97–13.79)*	0.23
BMI Waist Diet	1 (8, 3)	12 (0, 1)	0.94 (0.13–6.76)	
BMI Waist Inactivity	73 (28, 1)	260 (3, 2)	2.79 (2.08–3.74)*	2.06
Diet Smoke Inactivity	17 (12, 2)	139 (1, 7)	1.91 (1.14–3.18)*	0.82
Waist Smoke Inactivity	1 (20)	5 (0, 1)	2.21 (0.30–15.93)	
Waist Smoke Diet	0	1 (0, 01)		
4 MLFs	93 (29, 9)	311 (3, 8)		
BMI Diet Smoke Inactivity	6 (16, 7)	36 (0, 4	2.47 (1.08–5.61)*	0.26
BMI Waist Smoke Inactivity	61 (24, 4)	250 (3, 1)	2.72 (2.00–3.70)*	1.95
BMI Waist Diet Inactivity	3 (21, 4)	14 (0, 2)	2.15 (0.68–6.78)	
BMI Waist Diet Smoke	3 (30)	10 (0, 1)	4.30 (1.36–13.64)*	0.09
Waist Diet Smoke Inactivity	0	1 (0, 01)		
5 MLFs	10 (55, 6)	18 (/0, 2)		
Waist Diet BMI Smoke Inactivity	10 (55, 6)	18 (0, 2)	9.34 (4.85–17.99)*	0.20
Total	1, 046	8, 601		

aHR: HR adjusted for all the other combination of MLFs;

*p < 0,05.

Participants exposed to two MLFs were 2,315 women (28.6%) and 1,288 men (33%). The highest prevalence was observed for “smoking–inactivity” (11% women; 8% men), “BMI-smoking” (5% women; 13% men) and “BMI-inactivity”(6% women,men), with a HR (95% CI) of 1.19 (0.91–1.57), 1.53 (1.10–2.13), 2.04 (1.57–2.64) for women and 1.95 (1.14–3.35), 2.70 (1.66–4.42), 3.09 (1.82–5.25) for men, respectively. The highest impact was showed by “BMI-Inactivity” for women (PAR% 3.4), and by “BMI-Smoking” for men (PAR% 8.16).

Participants exposed to three MLFs were 998 women (12%) and 874 men (23%). The highest prevalence was observed for “BMI-smoking-inactivity” (4% women, 11% men), “waist-BMI-smoking” (2% women, 3% men), “waist-BMI-inactivity” (3% women,1% men) and “diet-smoking-inactivity”(1,7% women, 4% men), with a HR (95% CI) respectively of 2.17 (1.59–2.95), 3.09 (2.19–4.33), 2.79 (2.08–3.74), 1.91 (1.14–3.18) for women and of 3.13 (1.92–5.10), 7.18 (4.19–12.32), 3.85 (1.79–8.27), 2.15 (1.13–4.08) for men. The combination that showed the highest impact was “BMI-smoking-inactivity” (PAR% women 2.1; men 7.6). The combination of 3 MLFs at higher risk in women was “BMI-diet-smoking” with a HR (95% CI) of 6.40 (2.97–13.79).

Participants exposed to four MLFs were 311 women (4%) and 303 men (7.9%), with the highest prevalence showed by the combination of “BMI-waist-smoking-inactivity” (3.1% women, 3.5% men). This was also the combination at highest risk for men, with HR (95% CI) of 4.07 (2.35–7.05). For women, the combination at highest risk was “BMI-waist-diet-smoking” with a HR (95% CI) of 4.30 (1.36–13.64). The highest PAR% was shown by “BMI-waist-smoking-inactivity” (PAR% 1.9 women; 2.6 men). The simultaneous presence of all MLFs was detected only in 18 women (0.2%) and 33 men (0.9%), with a HR (95% CI) of 9.34 (4.85–17.99) for women and of 5.72 (2.71–12.08) for men.

### Clustering and interactions

Clustering was defined for a combination when its observed prevalence was different from the one expected assuming the component MLFs aggregate by chance. Hereby “cluster” is intended as a combination of MLFs that shows clustering. A total of 14 clusters (C) of MLFs (Table 5) for both women and men were identified, of which only 8 showed a notable association with the incidence of hypertension, consistent between sexes.

**Table 5:** Clustering and interactions of combined MLFs.

	Men				Women			
	Prevalence		HR		Prevalence		HR	
	Expected	O/E	Expected	O/E	Expected	O/E	Expected	O/E
**No MLF**	5.77	1.39	ref.		15.68	1.24	ref.	
**1 MLF**								
BMI	7.61	1	—	—	8.82	0.81	—	—
Diet	1.44	1.11	—	—	1.35	0.85	—	—
Smoking	10.94	1.1	—	—	12.76	1.18	—	—
Inactivity	4.87	1.08	—	—	12.77	0.95	—	—
**2 MLFs**								
BMI Inactivity	6.42	0.94	3.77	1.5	7.19	0.91	1.92	0.99
BMI Smoking	14.43	0.9	3.94	1.26	7.18	0.67	1.69	0.82
BMI Waist	0.92	1.23	2.43	1.04	1.25	2.12	4.34	0.94
BMI Diet	1.90	0.58	2.02	0.64	0.76	0.46	2.58	0.1
Inactivity Diet	1.21	0.95	1.29	1.72	1.10	1.35	1.56	0.89
Inactivity Smoking	9.23	0.91	2.51	1.4	10.40	1.09	1.02	0.99
Diet Smoking	2.73	1.1	1.34	1.33	1.10	1.21	1.37	0.78
**3 MLFs**								
BMI Smoking Inactivity	12.17	0.91	6.10	1.26	5.85	0.68	1.82	1.01
BMI Waist Smoking	1.74	1.65	3.94	2.11	1.01	2.41	4.12	1.16
BMI Diet Inactivity	1.60	0.71	3.13	1.88	0.61	0.79	2.78	0.98
BMI Diet Smoking	3.59	0.81	3.27	1.51	0.62	0.44	2.45	2.33
BMI Waist Diet	0.23	0.57	2.02	1.24	0.11	1.4	6.29	0.25
BMI Waist Inactivity	0.77	1.42	3.77	1.17	1.02	3.16	4.69	0.95
Diet Smoking Inactivity	2.30	1.58	2.08	1.51	0.16	1.1	1.48	1.1
**4 MLFs**								
BMI Diet Smoking Inactivity	3.03	1.09	5.06	1.17	0.50	0.87	2.64	0.78
BMI Waist Smoking Inactivity	1.47	2.41	6.10	1.03	0.83	3.73	4.45	0.89
BMI Waist Diet Inactivity	0.19	1.24	3.13	1.26	0.09	1.94	6.80	0.5
BMI Waist Diet Smoking	0.43	1.98	3.27	1.14	0.08	1.37	5.98	1.1
**5 MLFs**								
Waist Diet BMI Smoking Inactivity	0.37	2.35	5.06	1, 42	0.07	3.09	6.46	2.08

O/E = Observed/Expected ratio.

The most numerous clusters for women were “C-no risk” (19.5% of the female participants), “C-Smoking” (15%) and “C-Inactivity” (12%). For men, the most numerous clusters were “C-BMI-Smoking” (12.9% of the male participants), “C-Smoking” (12%) and “C-BMI-Smoking-Inactivity” (11%). In women, the clusters with the highest O/E prevalence ratios were “C-Waist-BMI-Smoking-Inactivity” (O/E prevalence = 3.73), “C-Waist-BMI-Diet-Smoking-Inactivity” (O/E prevalence = 3.09), and “C-Waist-BMI-Inactivity” (O/E prevalence = 3.16). In men the O/E prevalence ratios were generally lower, and the clusters with the highest ones were “C-Waist-BMI-Smoking-Inactivity” (O/E prevalence = 2.41), “C-Waist-BMI-Diet-Smoking-Inactivity” (O/E prevalence = 2.35) and “C-Waist-BMI-Diet-Smoking” (O/E prevalence = 1.98).

Among women, the most robust interaction was observed for “BMI-diet-smoking” (O/E HR: 2.33), followed by“C-Waist-BMI-Smoking” (O/E HR = 1.16). Interactions were slightly higher in men, and the highest O/E HR ratios were observed for “C-Waist-BMI-Smoking” (O/E HR = 2.11), “C-Waist-BMI-Diet-Smoking-Inactivity” “BMI-Diet-Inactivity”* (O/E HR = 1.88) and “Inactivity-Diet”* (O/E HR = 1.72) (Table 5). Notably, in women synergistic interactions were less frequent, and only 4 combinations of MFLs out of 25 showed an overlapping of clustering and interactions: “C-Waist-BMI-Smoking”, “C-Diet-Smoking-Inactivity”, “C-Waist-Diet-BMI-Smoking” and “C-Waist-BMI-Diet-Smoking-Inactivity”. In men, all the combinations of MLFs except for “BMI and diet”^1^* showed a O/E HR ratio >1, and an overlapping of clustering and interactions was observed in 10 of the 25 combinations (Table 5).

## Discussion

In the prospective EPIC-Potsdam cohort study, combinations of five out of six well recognised high-risk MLFs for hypertension such as high general adiposity, abdominal adiposity, smoking, low adherence to DASH diet and physical inactivity, were associated with an increased risk of incident hypertension during follow-up.

General and abdominal adiposity, smoking and physical inactivity appear to play a major role in increasing the risk of incidence of hypertension. However, only general adiposity appeared to be a sufficient cause to enhance the risk of hypertension per se. Moreover, these MLFs showed a high tendency to cluster in specific patterns, as in some case they showed an observed prevalence over 3 times higher than how expected by chance.

When single MLFs were investigated one by one in the whole cohort, general adiposity showed the most notable association with incidence of hypertension. Similar risk estimates had been found in a recent meta-analysis for this MLF^32^, which further confirms that general adiposity plays a central role for risk, regardless of differences in genetical, sociocultural and environmental backgrounds. Abdominal obesity was found to increase the risk of developing hypertension in both sexes, consistently with findings from other cohort studies^11,33^.

Adherence to DASH diet was not uniformly distributed between sexes, with a remarkably higher percentage of men in a high-risk category compared to women. In the first analysis, low adherence to DASH diet increased the risk of hypertension only among women. This was somehow unexpected, as to date the benefits of a DASH diet regime are well recognised for both primary and secondary prevention^19,34^ in women and men^34–37^. However, a few studies have found no or weak evidence that greater concordance with DASH diet had a long-term impact on BP change or hypertension over time^38,39^. In our study, the lack of association might be the result of the low number of men in the reference group that increases the probability of a false negative relation.

In this cohort, smoking appeared to be associated with higher risk of hypertension in men but not in women. Smoking has been largely recognised as an important risk factor of hypertension. It is associated with phenomena involved in hypertension’s pathogenesis, such as endothelial dysfunction^40^, atherosclerotic plaque formation^41^ or arterial stiffness^42^ and it showed to have a role in the development of hypertension in epidemiological studies^15,16,43,44^. However, our findings are not isolated. In a large study involving 33,860 people, it was shown that any independent chronic effect of smoking on BP is small, with a significantly higher value of SBP only in men smokers^45^. In a study involving 28,236 women, Bowman *et al*. have found that risk for hypertension increases by consuming at least 15 cigarettes per day, whereas non-significant association have been observed for less amount^46^. In the present study, we did not consider the numbers of cigarettes consumed per day, and this might have underestimated the effect of heavy smoking on the incidence of hypertension.

Physical inactivity showed a similar increase in risk for hypertension in both sexes and comparable values in terms of PAR%, suggesting that this MLF is associated with hypertension regardless of behavioural and biological attributes to sex. This is consistent with the major findings so far^47^.

An unexpected finding was that alcohol consumption at baseline seemed not to increase the risk of hypertension despite evidence from other studies stating the opposite^13,14^. We repeated the analyses using a “lifetime alcohol” variable^48^, and obtained the same result as with baseline alcohol intake (data not shown). After performing sensitivity analyses, the results did not change (data not shown). We argue with a couple of possible explanations. First, this might be possibly the result of low statistical power due to a too small number of people in the reference group of no/light consumers, especially in men. Second, it might be related to the genetic backgrounds of the participants involved in the studies. The dose-response curve for alcohol is not homogenous among studies^49,50^, and there is evidence that the effect of alcohol is strictly related to the enzymatic pattern of individuals^51–53^. It is known that the genetic background of individuals plays a role in shaping their susceptibility to lifestyle factors’ effect on hypertension development^54,55^. Specifically, it has been found that apolipoprotein E polymorphism influences the effect of alcohol on hypertension, conferring more or less proneness to blood pressure increase depending on the allele carried^56,57^.

The analysis of combined MLFs examined separately subgroups of participants characterised by a defined set of MLFs. This allowed us to disentangle their effective contribution to risk modulation and helped to provide with detailed insights regarding how the MLFs are distributed.

The analysis of the subgroups with only one MLF showed that general adiposity is the only risk factor which in our cohort constitutes by itself a sufficient cause of hypertension. All other MLFs did not show an association with the incidence of hypertension if they occurred individually. Also, this analysis revealed that both smoking and physical inactivity were more frequent in women compared to men when they occur as isolated MLF. This is in contradiction with the finding of the first analysis, where the overall prevalence of physical inactivity was almost equal between sexes and the prevalence of smoking was higher in men compared to women (Table 2). We could notice only two combinations without BMI that increased the risk of hypertension: “smoking and inactivity” and “smoking, inactivity and diet”, for both sexes. These combinations without BMI had a notable prevalence and were important in explaining the risk.

Among men, PARs% were generally higher compared to women. For example, “BMI-smoking” has a PAR% of 8.2 in men, whereas 1.7 in women, and when those two factors go along with physical inactivity PAR% became 7.6 for men and 2.1 for women. This could be the consequence of an overall healthier behaviour for women so that the range of improvement is wider for men. The percentage of smoking among women with one or more risk factors is remarkably lower than the one observed in men. From this evidence, it might be speculated that in men this risk factor is more likely to be combined with overweight and inactivity, while in women smoking is more likely to occur while having an otherwise healthy lifestyle.

General adiposity combined with low adherence to DASH diet was less prevalent than expected in both sexes, and this might indicate that in the present cohort, overweight and obese people tend to have more attention for their diet. Moreover, this combination was associated with a higher risk of hypertension in women but not in men. This might be again the consequence of low statistical power due to the small number of men in the reference group. General and abdominal adiposity showed to be aligned as it could have been expected and show a tendency to aggregate and being combined with smoking and inactivity, consistently across sexes. Regarding synergistic interactions, higher risk than expected varied by sex but the combination of general adiposity, smoking and diet showed higher risk than expected in both sexes.

This study has a number of strengths. It is based on a cohort of the EPIC study, characterised by a large number of participants, with the availability of data on multiple risk factors for hypertension. Data collection and measurements were highly standardised and carried with a rigorous methodology. To the best of our knowledge, this is the first study that takes into account details about the specific combinations of MLFs on hypertension risk, allowing to study the interrelation and the relative contribution of each MLF to the risk of hypertension. Moreover, the analysis of clustering of hypertension risk factors is of particular importance because it provides insights into how MLFs tend to aggregate.

Nevertheless, some limitations of this study also need to be examined. It should be considered that hypertension has been recognised as a polygenic disease with complexities such as ‘gene-gene’ and ‘environment-gene’ interactions. The MLFs may modulate in different ways the effect of genes on BP^54,55^, and we could not quantify the contribution of this interaction. Despite it is well recognised the role of salt in the onset of hypertension^58,59^, we could not consider it because the information on salt consumption was lacking. Thus we had to build a modified DASH index without taking it into account, and this might have underestimated the impact of diet on the risk of hypertension.

Further, the crude dichotomous categorisation made for calculating the sets of combinations and the PARs might underestimate the actual effect of the various risk factors. However, it has been demonstrated elsewhere that dichotomised lifestyle factors have been useful for studying the association of lifestyle factors and risk of diseases in large populations^60^.

The results of this study support the need to investigate MLFs by detailing the specific combinations of co-occurrence, as we found evidence that the traditional analysis of single MLFs by mutually adjusted models might be misleading due to a loss of crucial information. In the study MLFs showed a remarkable tendency to occur in specific patterns, strengthening the rational to promote a more comprehensive approach through multifactorial interventions which target more than a factor at a time. For prevention of hypertension addressing adiposity together with smoking and promoting physical activity should be the first choice.

## Data Availability

The datasets analysed during the current study are not publicly available due to data protection regulations. In accordance with German Federal and State data protection regulations, epidemiological data analyses of EPIC-Potsdam may be initiated upon an informal inquiry addressed to the secretariate of the Human Study Center (Office.HSZ@dife.de). Each request will then have to pass a formal process of application and review by the respective PI and a scientific board.

## Footnotes

1 **These combinations are not cluster*, *as their O*/*E prevalence ratio is* <*1*.
